# Fighting AMR in the Healthcare Environment: Microbiome-Based Sanitation Approaches and Monitoring Tools

**DOI:** 10.3390/ijms20071535

**Published:** 2019-03-27

**Authors:** Maria D’Accolti, Irene Soffritti, Sante Mazzacane, Elisabetta Caselli

**Affiliations:** 1 Section of Microbiology, Department of Medical Sciences, University of Ferrara, 44121 Ferrara, Italy; dccmra@unife.it (M.D.); sffrni@unife.it (I.S.); 2 CIAS Research Centre, Department of Architecture and Medical Science, University of Ferrara, 44121 Ferrara, Italy; mzs@unife.it

**Keywords:** healthcare-associated infections, antimicrobial resistance, sanitation, microbial technologies

## Abstract

Healthcare-associated infections (HAIs) affect up to 15% of all hospitalized patients, representing a global concern. Major causes include the persistent microbial contamination of hospital environment, and the growing antimicrobial-resistance (AMR) of HAI-associated microbes. The hospital environment represents in fact a reservoir of potential pathogens, continuously spread by healthcare personnel, visiting persons and hospitalized patients. The control of contamination has been so far addressed by the use of chemical-based sanitation procedures, which however have limitations, as testified by the persistence of contamination itself and by the growing AMR of hospital microbes. Here we review the results collected by a microbial-based sanitation system, inspired by the microbiome balance principles, in obtaining more effective control of microbial contamination and AMR. Whatever the sanitation system used, an important aspect of controlling AMR and HAIs relates to the ability to check any variation of a microbial population rapidly and effectively, thus effective monitoring procedures are also described.

## 1. Introduction

The discovery of penicillin in 1928 by Alexander Fleming, followed by continuous discovery and use of new antibiotics in the following decades, opened the so-called ‘antibiotic era’, where the management of bacteria-associated diseases was considered an obvious success, and bacterial infections as something no more representing a threaten for humans or animals.

Unfortunately, the massive use of antibiotics, exerting a continuous selective pressure, has led to the appearance and subsequent spread of mechanisms by which bacteria can survive to the antibiotic attack, ultimately leading to global and continuous growth of antimicrobial resistance (AMR) in many important human and animal pathogens.

Currently, this represents one of the most urgent threats to the public’s health, as AMR, initially limited to hospital strains (as they are exposed to the highest selective pressure), are now spreading rapidly to the general population.

AMR is particularly dangerous for hospital inpatients, as during hospitalization 5–15% of all hospitalized subjects acquire a so-called Healthcare Associated Infections (HAIs), which are mostly sustained by multidrug-resistant (MDR) or even pan-drug resistant microorganisms [1,2], rendering the therapeutic approach very difficult.

According to the European Centre for Disease Prevention and Control (ECDC), over 3 million patients contract an HAI in European acute hospitals every year, and 37,000 patients die as a direct HAI consequence [3,4]. In Italy HAI incidence is 5–10%, with infections caused by MDR microorganisms becoming more and more common, and with a mortality rate of 20–30% [5].

Based on several observations, the hospital environment plays an important role in HAI transmission, as it represents a reservoir of HAI-associated pathogens, which are easily spread by patients, visitors and hospital staffs [6]. For a long time hospital cleaning has been considered mostly an aesthetical requirement, but potential pathogens are not necessarily associated with evident dirt [7].

In fact, microorganisms have the ability to survive for long periods of time on surfaces [8], from where they can be easily transmitted to patients by direct or indirect contact [9]. Consistently with this, studies suggest a direct correlation between surface contamination and HAIs, as the risk to acquire a specific nosocomial pathogen increases when a patient is admitted in a room previously occupied by an infected or colonized patients by that specific infectious agent [10].

*Staphylococcus aureus*, *Enterobacteriaceae* (*Escherichia Coli*, *Klebsiella pneumoniae*), *Acinetobacter baumannii* and *Pseudomonas aeruginosa* are some of the most common HAI-related pathogens, also associated with AMR, which are known to persist for a long time in the hospital environment [6,8,11].

Among these species, *Staphylococcus aureus* is the one most commonly associated with bacteremia, infective endocarditis, skin and soft tissue infections [12], and has developed several resistances to multiple antibiotics in recent decades. Together with *Pseudomonas aeruginosa*, *Staphyloccocus aureus* represents a leading cause of nosocomial infections, mainly in compromised subjects [13,14]. Similarly, *Enterobacteriaceae* producing extended-spectrum β-lactamases and carbapenemases are continuously increasing, and their associated infections are often associated with high mortality [15].

Based on these observations, such microorganisms have been included in a global priority pathogen list (global PPL) of antibiotic-resistant bacteria, released in 2017 by the World Health Organization (WHO), where the methicillin-vancomycin resistant *Staphylococcus aureus* strain (MRSA, VRSA) is included in the “high” priority group and carbapenem-resistant *Enterobacteriaceae* and *Pseudomonas aeruginosa* have been included in the “critical” priority group [16].

## 2. Conventional and Alternative Sanitation Systems: Advantages and Disadvantages

Scientific evidence shows that the correct and appropriate cleanliness of hospital environments, together with campaign to raise awareness for hand hygiene, is accompanied by a reduction of surface contamination and a concomitant decrease of microbe transmission to patients [17,18].

Thus, the reduction of surface contamination appears a very important point toward control of MDR bacteria spread and management of nosocomial infections.

So far, decontamination of the hospital surfaces has been addressed by the use of chemicals-based detergents and disinfectants, and following specific timings [19].

However, recent studies show that more than 50% of surfaces result inadequately decontaminated when chemical products are used [20], and several microbes are persistently residing on treated surfaces, as judged by measurements of general markers [21] or specific search for individual pathogens [8,11,22]. Conventional sanitizers are in fact not able to prevent recontamination phenomena, which occur in continuum and are ultimately responsible for the persistence of surface contamination. Indeed, recontamination can occur as fast as 30 min after the application of chemical cleansers, leading to the reconstitution of the original contamination level and consequently keeping hospital inpatients in contact with potentially harmful pathogens for most of the time [23].

Even more importantly, chemical sanitation was shown to contribute to the selection of resistant strains [24]. Selected strains can become resistant not only toward the disinfectant itself but also, more importantly, against antimicrobial drugs, as recently reported for the disinfectant chlorhexidine, capable of inducing resistance against the antibiotic colistin [25]. Considering that colistin is used as a last-resort antimicrobial drug for treatment of carbapenemase-producing *Enterobacteriaceae*, this side-effect of some disinfectants appears particularly undesirable and dangerous. In addition, following the first isolation of an *mcr-1* plasmid-driven resistance against colistin in 2016 [26], it has been evidenced in up to 2% of clinical isolates [27], but we detected it with an unexpectedly high frequency in *Enterobacteriaceae* isolated from hospital surfaces, suggesting that this resistance is silently spreading among gram negative bacteria in the hospital environment [28].

Given the fast increase of MDR bacteria and the growing awareness about the importance of environmental contamination, several recent automatic cleaning systems have been developed with the aim to improve the quality of cleaning practices in the hospital environment [20], including “no-touch” technologies, use of ultraviolet (UV) light or hydrogen peroxide (HP) [29,30].

The UV light emitted by portable automated units are germicidal, and their effectiveness as potential decontaminating tools have been demonstrated against *C. difficile* spores, MRSA, and Vancomycin-resistance Enterococci (VRE) in hospital rooms [31]. However, the effects are highly dependent on several parameters, including time of exposure, the intensity of light and position of the lamp toward the irradiated surface. On the other hand, the HP system exists in different formulations (e.g., HP vapours and dry aerosols) and was shown to be effective against several microorganisms, such as multi resistant Gram-negative bacteria, including *Acinetobacter* spp. [32]. However, both these systems are difficultly sustainable from an economic point of view, require trained staff to use them, and although offering higher cleanliness compared to manual techniques, cannot substitute daily cleaning, since they are usable only during the period between patients discharge and new patients admission [33]. In consideration of this, UV irradiation is currently considered as an effective adjunct, and not as a stand-alone technology [34].

Other alternative systems are represented by the use of specific self-disinfecting surfaces, using metals like iron, copper and silver [35,36,37]. In fact, silver ions (Ag+) can bind to thiol groups, which are present in bacterial wall proteins, inactivating them. Similarly, copper can generate reactive oxygen species, which can damage microbial lipids, nucleic acids, and proteins, leading to cell death.

Light-activated antimicrobial coatings and germicide-impregnated materials can be also used. However, these methods are not suitable for all types of surfaces or settings, and indeed the observed reduction of surface contamination was around 10 to 100 folds compared to untreated surfaces [38].

Based on these observations, it is clear that there is an urgent need for sustainable effective methods of cleaning, able to reduce contamination in a stable way.

Interestingly, the term “sanitation” has been undergoing an important conceptual evolution in recent years. In fact, recent findings provided evidence that a drastic antimicrobial approach directed against the eradication of microbes, is doomed to failure [39]. This concept stems from the recent acquisitions on the human microbiome, where it is known that gut microbiota is associated with a multitude of health conditions affecting not only gut, but also distal organs [40], thanks to its essential role in the continuous fighting the colonization by pathogens. By contrast, its depletion (for example after a prolonged antibiotic therapy) can greatly favour the implant of potentially pathogenic microbes.

Similarly, the skin microbiota acts as a barrier against pathogens, due to the beneficial microbiota antagonizing the arrival of potential pathogens [41]; consistently with this, chlorhexidine-based disinfectants, by removing these beneficial microorganisms, could facilitate opportunistic pathogen colonization, like MRSA, thus exposing to a higher risk of contracting an infection, rather than protecting [42]. Therefore, the elimination of all microbial components, including the “healthy” one, seems to result in an excessive perturbation of the ecological network, with consequent deleterious effects on our body [43].

Translating these observations to the environmental level, a “super-sanitation” is likely not representing a solution for the pathogen contamination problem, and trying to manage the balance of microbial populations by restoring healthful communities instead “over-sterilizing” the environment, might have a greater potential for effectiveness.

**Figure 1:**
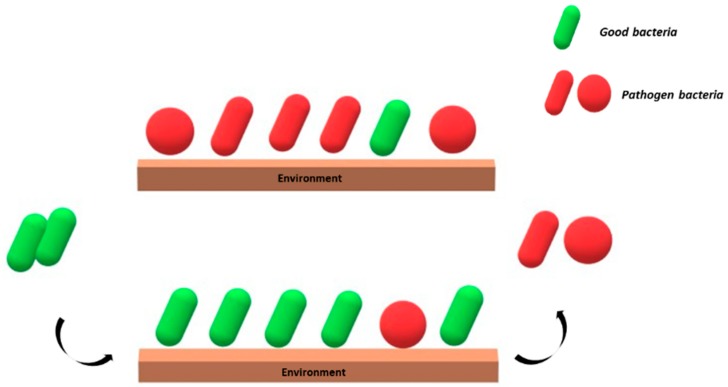
Schematic representation of the “Bygiene” principle. Good bacteria (green) introduced from the outside counteract the colonization of the environment by potential pathogens (red).

Al-Ghalith and Knights define this method “Bidirectional Hygiene, or Bygiene”, which consists of the introduction of beneficial species in the “environment”, which are able to counteract the colonization from pathogen microorganisms [42] (Figure 1).

## 3. Probiotics: An Overview

Among the microorganisms potentially useful toward sanitation aim, probiotics appear particularly interesting, as they are “live microorganisms that confer a health benefit to the host when administered in adequate amounts“, accordingly with FAO/WHO guidelines [44].

The original hypothesis of the positive role played by certain bacteria was first introduced in 1908 by the Russian scientist and Nobel Laureate Elie Metchnikoff, who observed that the consumption of fermented foods (particularly with lactic acid bacteria) would elicit beneficial effects on human health [45]. Since Metchnikoff’s original findings, several studies confirmed the positive role of probiotics on the health status of the host [46]. According to this, the use of probiotics has considerably increased in recent decades, and today there is considerable interest in probiotics for a variety of medical conditions, so much so that millions of people around the world consume them daily for perceived health benefits.

General criteria for probiotic selection include their safety in the host, ability to adhere to surfaces and to act by competitive antagonism against pathogenic bacteria [47,48]. Several probiotics used to restore the balance of the intestinal microbiota [49], are in fact acting through a “competitive exclusion” mechanism, that limit colonization by pathogens. Many evidence-based analyses from human studies and animal models have shown the clinical potential of probiotics against many pathogens [50]. According to this, their application in the clinical field for human disease treatment is very wide: Infectious diseases, diarrhoea, intestinal infections and inflammatory diseases are many examples [51,52]. Notably, probiotics have been also shown their efficiency in reducing the occurrence of important nosocomial infections, including upper respiratory infections [53], antibiotic-associated diarrhoea [54], and necrotizing enterocolitis [55].

## 4. *Bacillus*-Based Applications for Environmental Sanitation in Healthcare Settings

Aiming to approach the health of the environment as the health of the human/animal body, we recently studied an innovative cleaning system for hospital environment (Probiotic Cleaning Hygiene System, PCHS), based on the use of non-pathogenic probiotic bacteria belonging to the *Bacillus* genus (*B. subtilis*, *B. megaterium*, *B. pumilus*).

*Bacillus* bacteria are gram-positive, rod shaped, spore-forming, ubiquitous bacteria (they are present in soil, water, and the human gut), considered safe except for two well recognizable species (*Bacillus anthracis* and *Bacillus cereus*) [56,57,58].

*Bacillus* spores have a long history of safe application in humans, including: Food preparation (i.e., Natto, traditional Japanese food made from soybeans fermented with *Bacillus subtilis var. natto*), agriculture (i.e., Serenade, antifungal agent for food bio-preservation which consists of *Bacillus subtilis*, strain QST 713) [59], aquaculture [60,61], veterinary [62], human therapy in the gut [63] and pharmaceutical industry [64].

Some *Bacillus* strains have been shown to possess antimicrobial activity against various pathogens, as they can eradicate *S. aureus* by inhibiting its quorum sensing molecules [65], have an antagonistic effect against *Helicobacter pylori* [66] and *Campylobacter* spp. [67]. Furthermore, we recently observed that they can also inhibit the growth of environmental microbes contaminating ancient painting contaminants, suggesting a very generalizable use [68]. As spores are resistant to many physical-chemical factors, *Bacillus* are particularly suitable for addition to detergents, as they do not lose activity.

Consistently with these features, results collected by us and other groups, reported a great potential of *Bacillus*-based cleansers as sanitizing agents [23,69,70,71,72,73].

In particular, *Bacillus* spores, while preserving their viability in concentrated cleansers, can germinate when diluted in water and seeded on surfaces, originating the vegetative bacteria [70], able to inhibit almost completely the growth of both Gram-positive and Gram-negative bacteria, as well as of mycetes [23,70]. Although the main mechanism of action is based on competitive antagonism (Gause’s law) [74], they also produce antibacterial compounds that increase their antibacterial activity (bacteriocins), as suggested by the results obtained in vitro by stab overlay assays and inhibition growth tests using several indicator strains (personal unpublished results).

Studies performed in the hospital environment (analysing surface bioburden in three Italian and one Belgium hospitals), showed that PCHS usage induced a stable remodulation of the microbiota on treated surfaces, inducing a steady abatement of pathogen contamination up to 90% more than conventional disinfectants [23]. The effect was associated with the ability of *Bacillus* to compete with and replace pre-existing pathogens, arriving to represent about 70% of the total surface microbiota after only 1 month of application [70].

Most importantly, PCHS effect was not accompanied by any selection of drug resistant strain, but rather it decreased pathogen drug resistance genes harboured by the original pre-PCHS microbiota on treated surfaces, as measured by molecular and conventional assays [70,71,75].

In addition, specific studies were designed to evaluate their safety of use, being aware that PCHS *Bacillus* are alive microorganisms, although they are classified as non-pathogenic [76]. In particular, microbiological surveillance was implemented since 2011 in all treated hospitals, monitoring both genetic stability of PCHS-derived Bacilli and their infectious potential in hospitalized patients.

The results, did not evidence any acquisition of new resistance genes by PCHS *Bacillus* from the surrounding pathogens, suggesting a high genetic stability and the absence of mutagenicity or genetic exchange, despite the continuous contact with surface pathogenic and drug-resistant bacteria for long periods of time [70].

Similarly, the analysis of over 60,000 clinical specimens, derived from HAI or uninfected patients hospitalized in treated structures, evidenced the total absence of PCHS-*Bacillus*-positive samples, confirming that such *Bacillus* do not represent an infectious risk for hospitalized patients [71].

**Figure 2:**
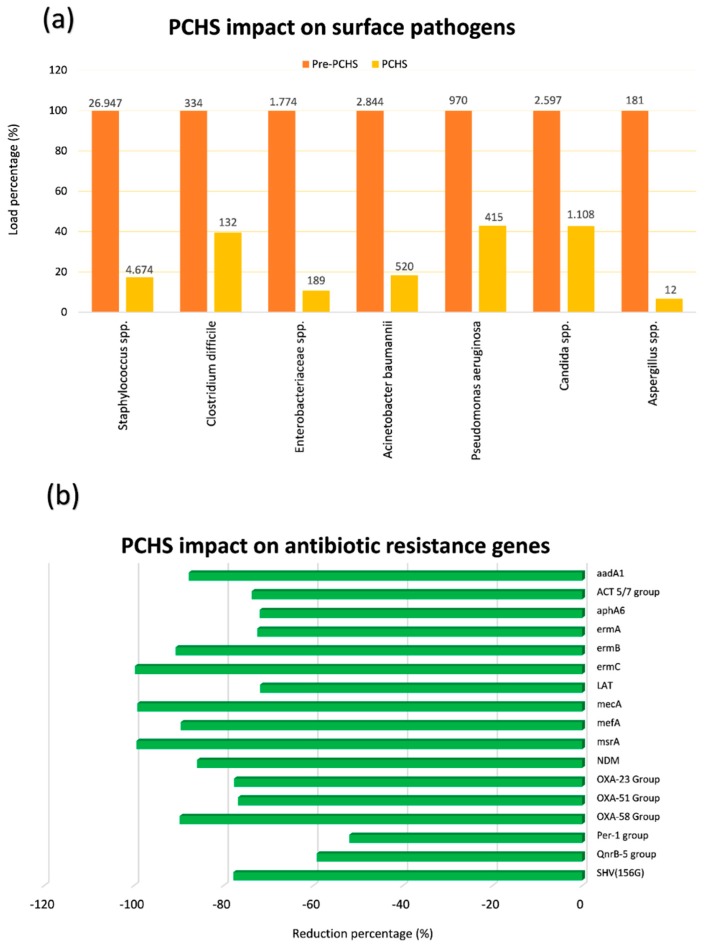
Probiotic Cleaning Hygiene System (PCHS) effect on microbial contamination and its antimicrobial-resistance (AMR) characteristics on hospital surfaces. (**a**) Six healthcare-associated infections (HAI)-associated pathogens were measured by Colony Forming Unit (CFU) count on hospital surfaces of five Italian hospitals before and after PCHS introduction; microbial load is expressed as percentage for each individual analyzed pathogen (mean values per m^2^ are also displayed); (**b**) the resistome of contaminating microbial population was analyzed by qPCR microarray before and after PCHS introduction; the most prevalent antibiotic-resistance genes are reported, and expressed as percentage reduction of genes during the PCHS phase compared to pre-PCHS phase.

Finally, a recent multicentre, pre-post interventional study performed in six public Italian hospitals for 18 months, showed that PCHS use was associated with a significant decrease of HAIs [73,77]. In fact, while bioburden analyses confirmed PCHS ability to remodulate the environmental contamination in all settings (Figure 2a) [23,70,73], as well as to decrease AMR in the contaminant microbial population (Figure 2b) [70,73,75], the continuous survey of HAI development evidenced a 52% reduction of HAI incidence in all the hospitals enrolled in the study [73].

In particular, in all treated healthcare structures, a persistent surface contamination was detected—mostly represented by Gram-positive bacteria belonging to the *Staphylococcus* genus, whereas other genera were less represented, although present. Interestingly, Gram-negative *Enterobacteriaceae* were generally little prevalent, evidencing a good level of cleaning in all enrolled structures. However, although cleaning conditions were generally good, a high level of Staphylococcal contamination was persistent observed, likely due to their continuous spread by patients and staff, and to their high resistance on inanimate surfaces [70,73]. Consistently with that, a high level of genes coding for methicillin resistance (*mecA*), typically present in *Staphylococcus* strains, was generally observed [70,73,75]. Other resistances were however well represented, although to a variable extent, likely depending on the selective pressure exerted by the specific antimicrobial use of each healthcare structure [75]. Notably, a daily cleaning by PCHS determined a clear reduction of the most prevalent contaminating microbes, up to 90% compared to conventional chemical-based cleaning, accompanied by a drop of the resistances usually associated with the individual microbial species (i.e., *mecA* driven methicillin resistance was reduced up to 99%). Consistently with resistome results, MDR species, as judged by conventional antibiograms, were reduced up to 75% compared to what observed during chemical cleaning [75]. The same strains resulted also reduced in HAI patients, who also needed less antimicrobial therapy, as suggested by the strong reduction of antimicrobial consumption and costs (60.3 and 75.4%, respectively).

These data strengthen the correlation between environmental contamination and HAI acquisition, suggesting that an effective cleaning intervention could affect significantly both AMR and HAI spread.

Of note, recent observations reported that the consumption of *Bacillus subtilis* spores was associated with eradication of *S. aureus* in a rural Thai population, this likely by blocking pathogen’s signalling, as shown in animal models [65]. The hypothesis that *Bacillus* intake might favour pathogens eradication from exposed/treated subjects is also supported by studies performed in animals, showing the ability of *B. subtilis* and *B. licheniformis* to modify the intestinal microbiota structure in chickens, thus normalizing the disorders caused by necrotic enteritis in chickens [78,79]. These observations suggest that similar alterations might occur in PCHS-exposed patients, ultimately protecting them from infections, and highlight the need for further studies on the interaction of PCHS with patient’s microbiome, which could important in understanding the mechanism by which PCHS protects from HAIs.

## 5. Analyses Tools

Although conventional microbiology has been of immense value for many years, it shows limitations, linked to a need for cultural isolation, culture time, biochemical identification, etc.

In fact, although having the advantage of evidencing only alive microbes, culture-based methods need from 48 to 120 h to detect searched microbes; moreover some microorganisms are difficult to grow, need specific culture media and particular growth conditions (as to temperature and time of incubation, or anaerobiosis conditions), or grow with different efficiency, thus rendering very time-consuming and complex the analysis of a whole microbial population.

Similarly, assays based on the measurement of adenosine triphosphate (ATP) by bioluminescence assay, commonly used for assessing the effectiveness of cleaning procedures, even providing results rapidly, but they are not accurate, identifying not only alive microbes on surfaces but also organic materials, and being further influenced by residues of detergents/disinfectants [80].

By contrast, an efficient monitoring system should be able to provide rapid and precise information on the microbial population examined, leading to a detailed characterization of the environmental bioburden in real time.

Molecular methods, based on the DNA technologies, can consistently help in overcoming such limitations, providing simultaneous analysis of high numbers of different parameters, and thus helping to precisely define the microbial populations from both a qualitative and quantitative point of view. These features, together with the rapidity of analysis compared to conventional methods, can improve substantially the analysis efficiency, provide evidence of unfavourable events, and encourage immediate countermeasures. Consistent with this, in recent years, many molecular assays have been developed and commercialized, and we too set up and implemented several molecular analyses in our studies, comparing them with conventional microbiological methods, when possible, to validate the developed molecular assays [23,70,71,73,75].

One of the main advantages of molecular assays with respect to conventional microbiology consist in their high rapidity, sensitivity and specificity, thanks to which even a very low number of targets can be efficiently detected in few hours. This aspect is particularly useful when it is needed to evidence even minimal variations in the composition of a complex microbial population, allowing to obtain rapid results without depending on culture isolation, and ultimately being extremely powerful to monitor surface bioburden, as well as clinical isolates. Both qualitative polymerase chain reactions (PCR), quantitative real time PCR and qPCR microarrays have been proven to be effective tools toward this aim. Such methods are currently available at affordable costs and an updated microbiology laboratory might include these analysis methodologies, as they can provide details otherwise impossible to detect by conventional tests. In particular, we applied PCR and qPCR assays to identify and quantify *Bacillus* strains qPCR, to amplify and quantify the total bacterial or mycetes load, and to detect and quantify specific pathogens or resistance genes [23,70,71,73,75]. qPCR microarrays in particular provided us the chance to evaluate simultaneously the presence of over 80 resistance genes in a microbial sample, allowing to define the “resistome” of the whole contaminant microbiota, thus permitting to monitor the antibiotic-resistance profile of the entire population in a very accurate and timely way, compared to conventional antibiograms performed on individual isolates.

In addition to home-made assays, mostly used for research purposes, many other are now commercialized and certified for diagnosis, and can represent a critical advantage in monitoring the presence of specific pathogens and resistances within a contaminant population [81,82,83,84].

Last, the new techniques based on DNA sequencing, such as Next Generation Sequencing (NGS) and Whole Genome Sequencing (WGS), are currently starting to be a landmark for microbiome analyses, due to their capacity of defining complex populations in deep detail. The striking advantage of such methods, even compared to other molecular techniques, consists in the ability to analyse the whole population, not only the searched targets, thus offering a very complete picture of the microbial sample. Thanks to this feature, they might become an invaluable tool to monitor microbial populations contaminating hospital environment (similarly to what is done for human or animal microbiome). Preliminary data obtained by us in different settings are very promising, allowing to obtain precious information about the evolution of the microbiome characteristics and therefore to take consequent rapid countermeasures in case of adverse events.

## 6. Conclusions and Future Perspectives

Taken together, these results show that it is possible to counteract microbial contamination and AMR spread by a sustainable biological system, suggesting its potential use to manage the environmental hygiene in hospitals, and help to reduce the risk of infections in patients. Future studies on the eventual impact of a probiotic-based approach on the individual microbiome of hospitalized patients will be interesting, to understand and characterize in more detail the protective effect observed toward the development of HAIs.

Moreover, whatever the type of sanitation adopted, the set-up of very sensitive and precise molecular assays, to study and characterize microbial communities, could be important for monitoring microbial contamination and its evolution both in hospital environment and inpatients. The development of alternative methodologies which do not need culture isolation for the study of microorganisms, might in fact bring evident advantages in terms of precision and rapidity of response, allowing to define in detail the study microbial population, detect unfavourable events and take immediate countermeasures.

In conclusion, these data might be important for the development of future guidelines for hospital cleaning directed to prevention strategies to reduce infectious diseases onset, likely applicable not only in healthcare settings.

## References

[1] Caini S., Hajdu A., Kurcz A., Böröcz K. (2013). Hospital-acquired infections due to multidrug-resistant organisms in Hungary, 2005–2010. Eurosurveillance.

[2] Cornejo-Juárez P., Vilar-Compte D., Pérez-Jiménez C., Ñamendys-Silva S.A., Sandoval-Hernández S., Volkow-Fernández P. (2015). The impact of hospital-acquired infections with multidrug-resistant bacteria in an oncology intensive care unit. Int. J. Infect. Dis..

[3] Brusaferro S., Arnoldo L., Cattani G., Fabbro E., Cookson B., Gallagher R., Hartemann P., Holt J., Kalenic S., Popp W. (2015). Harmonizing and supporting infection control training in Europe. J. Hosp. Infect..

[4] European Centre for Disease Prevention and Control Annual Epidemiological Report 2013. https://ecdc.europa.eu/en/publications-data/annual-epidemiological-report-2013-2011-data.

[5] Messineo A., Marsella L.T. (2015). Biological hazards and healthcare-associated infections in Italian healthcare facilities: Some considerations on inspections and accountability. Ann. Ig..

[6] Otter J.A., Yezli S., French G.L. (2011). The role played by contaminated surfaces in the trasmission of nosocomial pathogens. Infect. Control Hosp. Epidemiol..

[7] Dancer S.J. (2009). The role of environmental cleaning in the control of hospital-acquired infection. J. Hosp. Infect..

[8] Kramer A., Schwebke I., Kampf G. (2006). How long do nosocomial pathogens persist on inanimate surfaces? A systematic review. BMC Infect. Dis..

[9] Otter J.A., Yezli S., Salkeld J.A., French G.L. (2013). Evidence that contaminated surfaces contributed to the transmission of hospital pathogens and an overview of strategies to address contaminated surfaces in hospital settings. Am. J. Infect. Control.

[10] Huang S.S., Datta R., Platt R. (2006). Risk of acquiring antibiotic resistant bacteria from prior occupants. Arch. Intern. Med..

[11] Boyce J.M. (2007). Environmental contamination makes an important contribution to hospital infection. J. Hosp. Infect..

[12] Diekema D.J., Pfaller M.A., Schmitz F.J., Smayevsky J., Bell J., Jones R.N., Beach M., SENTRY Participants Group (2001). Survey of Infections Due To Staphylococcus Species: Frequency of Occurrence and Antimicrobial Susceptibility of Isolates Collected in the United States, Canada, Latin America, Europe and the Western Pacific Region for the Sentry Antimicrobial Surveillance. Clin. Infect. Dis..

[13] Deleo F.R., Chambers H.F. (2009). Reemergence of antibiotic-resistant Staphylococcus aureus in the genomics era. J. Clin. Investig..

[14] Senthilkumar A., Kumar S., Sheagren J.N. (2001). Increased Incidence of Staphylococcus aureus Bacteremia in Hospitalized Patients with Acquired Immunodeficiency Syndrome. Clin. Infect. Dis..

[15] Palacios-Baena Z.R., Gutiérrez-Gutiérrez B., De Cueto M., Viale P., Venditti M., Hernández-Torres A., Oliver A., Martínez-Martínez L., Calbo E., Pintado V. (2017). Development and validation of the INCREMENT-ESBL predictive score for mortality in patients with bloodstream infections due to extended-spectrum-β-lactamase-producing Enterobacteriaceae. J. Antimicrob. Chemother..

[16] Tacconelli E., Magrini N., Carmeli Y., Harbarth S., Kahlmeter G., Kluytmans J., Mendelson M., Pulcini C., Singh N., Theuretzbacher U. Global Priority List of Antibiotic-Resistant Bacteria to Guide Research, Discovery and Development of New Antibiotics. https://www.who.int/medicines/publications/WHO-PPL-Short_Summary_25Feb-ET_NM_WHO.pdf.

[17] Dancer S.J. (2008). Importance of the environment in methicillin resistant Staphylococcus aureus acquisition: The case for hospital cleaning. Lancet Infect. Dis..

[18] Rampling A., Wiseman S., Davis L., Hyett A.P., Walbridge A.N., Payne G.C., Cornaby A.J. (2001). Evidence that hospital hygiene is important in the control of methicillin resistant Staphylococcus aureus. J. Hosp. Infect..

[19] National Patient Safety Agency (2007). The National Specifications for Cleanliness in the NHS: A Framework for Setting and Measuring Performance Outcomes. https://www.rdehospital.nhs.uk/docs/patients/services/housekeeping_services/2007NationalSpecforcleanlinessintheNHS.pdf%0D.

[20] Carling P.C., Parry M.F., Von Beheren S.M., Healthcare Environmental Hygiene Study Group (2008). Identifying opportunities to enhance environmental cleaning in 23 acute care hospitals. Infect. Control Hosp. Epidemiol..

[21] Goodman E.R., Piatt R., Bass R., Onderdonk A.B., Yokoe D.S., Huang S.S. (2008). Impact of an Environmental Cleaning Intervention on the Presence of Methicillin-Resistant Staphylococcus aureus and Vancomycin-Resistant Enterococci on Surfaces in Intensive Care Unit Rooms. Infect. Control Hosp. Epidemiol..

[22] Lawley T.D., Clare S., Deakin L.J., Goulding D., Yen J.L., Raisen C., Brandt C., Lovell J., Cooke F., Clark T.G. (2010). Use of purified clostridium difficile spores to facilitate evaluation of health care disinfection regimens. Appl. Environ. Microbiol..

[23] Vandini A., Temmerman R., Frabetti A., Caselli E., Antonioli P., Balboni P.G., Platano D., Branchini A., Mazzacane S. (2014). Hard surface biocontrol in hospitals using microbial-based cleaning products. PLoS ONE.

[24] Bock L.J., Wand M.E., Sutton J.M. (2016). Varying activity of chlorhexidine-based disinfectants against Klebsiella pneumoniae clinical isolates and adapted strains. J. Hosp. Infect..

[25] Wand M.E., Bock L.J., Bonney L.C., Sutton J.M. (2017). Mechanisms of Increased Resistance to Chlorhexidine and Cross-resistance to Colistin following Exposure of Klebsiella pneumoniae Clinical Isolates to Chlorhexidine. Antimicrob. Agents Chemother..

[26] Liu Y.Y., Wang Y., Walsh T.R., Yi L.X., Zhang R., Spencer J., Doi Y., Tian G., Dong B., Huang X. (2016). Emergence of plasmid-mediated colistin resistance mechanism MCR-1 in animals and human beings in China: A microbiological and molecular biological study. Lancet Infect. Dis..

[27] Ye H., Li Y., Li Z., Gao R., Zhang H., Wen R., Gao G.F., Hu Q. (2016). Diversified mcr-1-Harbouring Plasmid Reservoirs Confer Resistance to Colistin in Human Gut Microbiota. MBio.

[28] Caselli E., D’Accolti M., Soffritti I., Piffanelli M., Mazzacane S. (2018). Spread of mcr-1-Driven Colistin Resistance on Hospital Surfaces, Italy. Emerg. Infect. Dis..

[29] Weber D.J., Kanamori H., Rutala W.A. (2016). “No touch” technologies for environmental decontamination: Focus on ultraviolet devices and hydrogen peroxide systems. Curr. Opin. Infect. Dis..

[30] Boyce J.M. (2016). Modern technologies for improving cleaning and disinfection of environmental surfaces in hospitals. Antimicrob. Resist. Infect. Control.

[31] Nerandzic M.M., Cadnum J.L., Pultz M.J., Donskey C.J. (2010). Evaluation of an automated ultraviolet radiation device for decontamination of Clostridium difficile and other healthcare-associated pathogens in hospital rooms. BMC Infect. Dis..

[32] Chmielarczyk A., Higgins P.G., Wojkowska-Mach J., Synowiec E., Zander E., Romaniszyn D., Gosiewski T., Seifert H., Heczko P., Bulanda M. (2012). Control of an outbreak of Acinetobacter baumannii infections using vaporized hydrogen peroxide. J. Hosp. Infect..

[33] Dancer S.J. (2014). Controlling hospital-acquired infection: Focus on the role of the environment and new technologies for decontamination. Clin. Microbiol. Rev..

[34] Memarzadeh F., Olmsted R.N., Bartley J.M. (2010). Applications of ultraviolet germicidal irradiation disinfection in health care facilities: Effective adjunct, but not stand-alone technology. Am. J. Infect. Control.

[35] Noyce J.O., Michels H., Keevil C.W. (2006). Potential use of copper surfaces to reduce survival of epidemic meticillin-resistant Staphylococcus aureus in the healthcare environment. J. Hosp. Infect..

[36] Casey A.L., Adams D., Karpanen T.J., Lambert P.A., Cookson B.D., Nightingale P., Miruszenko L., Shillam R., Christian P., Elliott T.S.J. (2010). Role of copper in reducing hospital environment contamination. J. Hosp. Infect..

[37] Lansdown A.B.G. (2006). Silver in health care: Antimicrobial effects and safety in use. Curr. Probl. Dermatol..

[38] Weber D.J., Rutala W.A. (2013). Self-disinfecting surfaces: Review of current methodologies and future prospects. Am. J. Infect. Control.

[39] Vangay P., Ward T., Gerber J.S., Knights D. (2015). Antibiotics, pediatric dysbiosis, and disease. Cell Host Microbe.

[40] Shreiner A.B., Kao J.Y., Young V.B. (2015). The gut microbiome in health and in disease. Curr. Opin. Gastroenterol..

[41] Liu C.M., Price L.B., Hungate B.A., Abraham A.G., Larsen L.A., Christensen K., Stegger M., Skov R., Andersen P.S. (2015). Staphylococcus aureus and the ecology of the nasal microbiome. Sci. Adv..

[42] Al-Ghalith G.A., Knights D. (2015). Focus: Personalized Medicine: Bygiene: The New Paradigm of Bidirectional Hygiene. Yale J. Biol. Med..

[43] Ianiro G., Bibbò S., Gasbarrini A., Cammarota G. (2014). Therapeutic Modulation of Gut Microbiota: Current Clinical Applications and Future Perspectives. Curr. Drug Targets.

[44] FAO/WHO (2002). Guidelines for the Evaluation of Probiotics in Food.

[45] Vaughan R.B. (1965). The Romantic Rationalist a Study of Elie Metchnikoff. Med. Hist..

[46] Fernandes C.F., Shahani K.M., Amer M.A. (1987). Therapeutic role of dietary lactobacilli and lactobacillic fermented dairy products. FEMS Microbiol. Rev..

[47] Collins J.K., Thornton G., Sullivan G.O. (1988). Selection of Probiotic Strains for Human Application. Int. Dairy J..

[48] Ouwehand A.C., Salminen S., Isolauri E. (2002). Probiotics: An overview of beneficial effects. Antonie Van Leeuwenhoek.

[49] Isolauri E., Salminen S., Ouwehand A.C. (2004). Microbial-gut interactions in health and disease. Probiotics. Best Pract. Res. Clin. Gastroenterol..

[50] Fang Y., Polk D.B. (2011). Probiotics and immune health. Curr. Opin. Gastroenterol..

[51] Chen C.C., Walker W.A. (2005). Probiotics and prebiotics: Role in clinical disease states. Adv. Pediatr..

[52] Sullivan A., Nord C.E. (2002). The place of probiotics in human intestinal infections. Int. J. Antimicrob. Agents.

[53] Banupriya B., Biswal N., Srinivasaraghavan R., Narayanan P., Mandal J. (2015). Probiotic prophylaxis to prevent ventilator associated pneumonia (VAP) in children on mechanical ventilation: An open-label randomized controlled trial. Intensive Care Med..

[54] Squellati R. (2018). Evidence-Based Practice in the Treatment for Antibiotic-Associated Diarrhea in the Intensive Care Unit. Crit. Care Nurs. Clin. N. Am..

[55] Patel R.M., Underwood M.A. (2018). Probiotics and necrotizing enterocolitis. Semin. Pediatr. Surg..

[56] (2005). Opinion of the Scientific Panel on biological hazards (BIOHAZ) on Bacillus cereus and other Bacillus spp in foodstuffs. EFSA J..

[57] Goel A.K. (2015). Anthrax: A disease of biowarfare and public health importance. World J. Clin. Cases.

[58] Granum P.E., Lund T. (1997). Bacillus cereus and its food poisoning toxins. FEMS Microbiol. Lett..

[59] Leyva Salas M., Mounier J., Valence F., Coton M., Thierry A., Coton E. (2017). Antifungal Microbial Agents for Food Biopreservation—A Review. Microorganisms.

[60] Vaseeharan B., Ramasamy P. (2003). Control of pathogenic Vibrio spp. by Bacillus subtilis BT23, a possible probiotic treatment for black tiger shrimp Penaeus monodon. Lett. Appl. Microbiol..

[61] Liu C.-H., Wu K., Chu T.-W., Wu T.-M. (2018). Dietary supplementation of probiotic, *Bacillus subtilis* E20, enhances the growth performance and disease resistance against *Vibrio alginolyticus* in parrot fish (*Oplegnathus fasciatus*). Aquac. Int..

[62] Cutting S.M. (2011). Bacillus probiotics. Food Microbiol..

[63] Mazza P. (1994). The use of Bacillus subtilis as an antidiarrhoeal microorganism. Boll. Chim. Farm..

[64] Ripert G., Racedo S.M., Elie A.M., Jacquot C., Bressollier P., Urdaci M.C. (2016). Secreted compounds of the probiotic Bacillus clausii strain O/C inhibit the cytotoxic effects induced by Clostridium difficile and Bacillus cereus toxins. Antimicrob. Agents Chemother..

[65] Piewngam P., Zheng Y., Ngueyen T.H., Dickey S.W., Joo H.-S., Villaruz A.E., Glose K.A., Fisher E.L., Hunt R.L., Li B. (2018). Pathogen elimination by probiotic Bacillus via signalling interference. Nature.

[66] Pinchuk I.V., Bressollier P., Verneuil B., Fenet B., Sorokulova I.B., Mégraud F., Urdaci M.C. (2001). In vitro anti-Helicobacter pylori activity of the probiotic strain Bacillus subtilis 3 is due to secretion of antibiotics. Antimicrob. Agents Chemother..

[67] Sorokulova I.B., Kirik D.L., Pinchuk I.V. (1997). Probiotics against Campylobacter Pathogens. J. Travel Med..

[68] Caselli E., Pancaldi S., Baldisserotto C., Petrucci F., Impallaria A., Volpe L., D’Accolti M., Soffritti I., Coccagna M., Sassu G. (2018). Characterization of biodegradation in a 17 th century easel painting and potential for a biological approach. PLoS ONE.

[69] La Fauci V., Costa G.B., Anastasi F., Facciolà A., Grillo O.C., Squeri R. (2015). An Innovative Approach to Hospital Sanitization Using Probiotics: In Vitro and Field Trials. J. Microb. Biochem. Technol..

[70] Caselli E., D’Accolti M., Vandini A., Lanzoni L., Camerada M.T., Coccagna M., Branchini A., Antonioli P., Balboni P.G., Di Luca D. (2016). Impact of a probiotic-based cleaning intervention on the microbiota ecosystem of the hospital surfaces: Focus on the resistome remodulation. PLoS ONE.

[71] Caselli E., Antonioli P., Mazzacane S. (2016). Safety of probiotics used for hospital environmental sanitation. J. Hosp. Infect..

[72] Caselli E. (2017). Hygiene: Microbial strategies to reduce pathogens and drug resistance in clinical settings. Microb. Biotechnol..

[73] Caselli E., Brusaferro S., Coccagna M., Arnoldo L., Berloco F., Antonioli P., Tarricone R., Pelissero G., Nola S., La Fauci V. (2018). Reducing healthcare-associated infections incidence by a probiotic-based sanitation system: A multicentre, prospective, intervention study. PLoS ONE.

[74] Gause G. (1932). Experimental studies on the struggle for existence. J. Exp. Biol..

[75] Caselli E., Arnoldo L., Rognoni C., D’Accolti M., Soffritti I., Lanzoni L., Bisi M., Tarricone R., Brusaferro S., Mazzacane S. (2019). Impact of a probiotic-based hospital sanitation on antimicrobial resistance and HAI-associated antimicrobial consumption and costs: A multi-center study. Infect. Drug Resist..

[76] European Food Safety Authority (EFSA) Scientific Opinion on the Maintenance of the List of QPS Biological Agents Intentionally Added to Food and Feed (2010 Update). www.efsa.europa.eu/efsajournal.htm.

[77] Caselli E., Berloco F., Tognon L., Villone G., La Fauci V., Nola S., Antonioli P., Coccagna M., Balboni P.G., Pelissero G. (2016). Influence of Sanitizing Methods on Healthcare-Associated Infections Onset: A Multicentre, Randomized, Controlled Pre-Post Interventional Study. J. Clin. Trials.

[78] Ma Y., Wang W., Zh H., Wang J., Zhang W., Gao J. (2018). Supplemental Bacillus subtilis DSM 32315 manipulates intestinal structure and microbial composition in broiler chickens. Sci. Rep..

[79] Xu S., Lin Y., Zeng D., Zhou M., Zeng Y., Wang H. (2018). Bacillus licheniformis normalize the ileum microbiota of chickens infected with necrotic enteritis. Sci. Rep..

[80] Nante N., Ceriale E., Messina G., Lenzi D., Manzi P. (2017). Effectiveness of ATP bioluminescence to assess hospital cleaning: A review. J. Prev. Med. Hyg..

[81] QIAGEN Custom Microbial qPCR Arrays. https://www.qiagen.com/it/errors/500/?aspxerrorpath=/it/search/custom-microbial-dna-qpcr-arrays/&akamai-feo=off.

[82] Genesig Products Hub. http://www.genesig.com/products-hub.

[83] BIO-RAD Food PCR Testing Kits. http://www.bio-rad.com/en-us/category/food-pcr-testing-kits?ID=a83b4db2-1f52-4af9-babf-275e6509eb57.

[84] R-Biopharm AG RIDA^®^GENE Hospital Stool Panel. https://clinical.r-biopharm.com/products/ridagene-hospital-stool-panel/.

